# COVID-19 vaccination-related delayed adverse events among people with rheumatoid arthritis: results from the international COVAD survey

**DOI:** 10.1007/s00296-024-05742-x

**Published:** 2024-11-06

**Authors:** Mrinalini Dey, Bohdana Doskaliuk, Ioannis Parodis, Julius Lindblom, Chris Wincup, Mrudula Joshi, Dzifa Dey, Wanruchada Katchamart, Esha Kadam, Parikshit Sen, Samuel Katsuyuki Shinjo, Arvind Nune, Phonpen Akarawatcharangura Goo, Nelly Ziade, Yi Ming Chen, Lisa S. Traboco, Carlos Enrique Toro Gutiérrez, Binit Vaidya, Vikas Agarwal, Latika Gupta, Elena Nikiphorou, COVAD Study Group, Nelly Ziade, R Naveen, Sreoshy Saha, Ai Lyn Tan, Masataka Kuwana, Akira Yoshida, Keina Yomono, John D. Pauling, Ashima Makol, Jessica Day, Tulika Chatterjee, Lorenzo Cavagna, Vishwesh Agarwal, Marcin Milchert, Oliver Distler, Hector Chinoy, Tsvetelina Valikova, Abraham Edgar Gracia-Ramos, Johannes Knitza, Aarat Patel, Bhupen Barman, Erick Adrian Zamora Tehozol, Jorge Rojas Serrano, Ignacio García-De La Torre, Iris J. Colunga-Pedraza, Javier Merayo-Chalico, Okwara Celestine Chibuzo, Russka Shumnalieva, Leonardo Santos Hoff, Lina Kibbi, Hussein Halabi, A. T. M Tanveer Hasan, Carlo V. Caballero-Uribe, James B. Lilleker, Babur Salim, Tamer Gheita, Miguel A. Saavedra, Sinan Kardes, Laura Andreoli, Daniele Lini, Karen Schreiber, Melinda Nagy Vince, Yogesh Preet Singh, Rajiv Ranjan, Avinash Jain, Sapan C. Pandya, Rakesh Kumar Pilania, Aman Sharma, M. Manesh Manoj, Vikas Gupta, Chengappa G. Kavadichanda, Pradeepta Sekhar Patro, Sajal Ajmani, Sanat Phatak, Rudra Prosad Goswami, Abhra Chandra Chowdhury, Ashish Jacob Mathew, Padnamabha Shenoy, Ajay Asranna, Keerthi Talari Bommakanti, Anuj Shukla, Arunkumar R. Pande, Kunal Chandwar, Akanksha Ghodke, Hiya Boro, Zoha Zahid Fazal, Döndü Üsküdar Cansu, Reşit Yıldırım, Armen Yuri Gasparyan, Nicoletta Papa, Gianluca Sambataro, Atzeni Fabiola, Marcello Govoni, Simone Parisi, Elena Bartoloni Bocci, Gian Domenico Sebastiani, Enrico Fusaro, Marco Sebastiani, Luca Quartuccio, Franco Franceschini, Pier Paolo Sainaghi, Giovanni Orsolini, Rossella Angelis, Maria Giovanna Danielli, Vincenzo Venerito, Silvia Grignaschi, Alessandro Giollo, Alessia Alluno, Florenzo Ioannone, Marco Fornaro, Suryo Anggoro Kusumo Wibowo, Jesús Loarce-Martos, Sergio Prieto-González, Raquel Aranega Gonzalez, Ran Nakashima, Shinji Sato, Naoki Kimura, Yuko Kaneko, Takahisa Gono, Stylianos Tomaras, Fabian Nikolai Proft, Marie-Therese Holzer, Margarita Aleksandrovna Gromova, Or Aharonov, Zoltán Griger, Ihsane Hmamouchi, Imane bouchti, Zineb Baba, Margherita Giannini, François Maurier, Julien Campagne, Alain Meyer, Daman Langguth, Vidya Limaye, Merrilee Needham, Nilesh Srivastav, Marie Hudson, Océane Landon-Cardinal, Wilmer Gerardo Rojas Zuleta, Álvaro Arbeláez, Javier Cajas, José António Pereira Silva, João Eurico Fonseca, Olena Zimba, Doskaliuk Bohdana, Uyi Ima-Edomwonyi, Ibukunoluwa Dedeke, Emorinken Airenakho, Nwankwo Henry Madu, Abubakar Yerima, Hakeem Olaosebikan, A. Becky, Oruma Devi Koussougbo, Elisa Palalane, Ho So, Manuel Francisco Ugarte-Gil, Lyn Chinchay, José Proaño Bernaola, Victorio Pimentel, Hanan Mohammed Fathi, Reem Hamdy A. Mohammed, Ghita Harifi, Yurilís Fuentes-Silva, Karoll Cabriza, Jonathan Losanto, Nelly Colaman, Antonio Cachafeiro-Vilar, Generoso Guerra Bautista, Enrique Julio Giraldo Ho, Lilith Stange Nunez, Vergara M. Cristian, Jossiell Then Báez, Hugo Alonzo, Carlos Benito Santiago Pastelin, Rodrigo García Salinas, Alejandro Quiñónez Obiols, Nilmo Chávez, Andrea Bran Ordóñez, Gil Alberto Reyes Llerena, Radames Sierra-Zorita, Dina Arrieta, Eduardo Romero Hidalgo, Ricardo Saenz, Escalante M. Idania, Wendy Calapaqui, Ivonne Quezada, Gabriela Arredondo

**Affiliations:** 1 Centre for Rheumatic Diseases, King’s College London, London, UK; 2 Department of Pathophysiology, Ivano-Frankivsk National Medical University, Ivano-Frankivsk, Ukraine; 3 Division of Rheumatology, Department of Medicine Solna, Karolinska Institutet and Karolinska University Hospital, Stockholm, Sweden; 4 Department of Rheumatology, Faculty of Medicine and Health, Örebro University, Örebro, Sweden; 5 Rheumatology Department, King’s College Hospital, London, UK; 6 Byramjee Jeejeebhoy Government Medical College and Sassoon General Hospitals, Pune, India; 7 Department of Medicine and Therapeutics, Rheumatology Unit, University of Ghana Medical School, College of Health Sciences, Korle-Bu, Accra, Ghana; 8 Division of Rheumatology, Department of Medicine, Faculty of Medicine Siriraj Hospital, Mahidol University, Bangkok, Thailand; 9 Seth Gordhandhas Sunderdas Medical College and King Edwards Memorial Hospital, Mumbai, Maharashtra India; 10 Maulana Azad Medical College, 2-Bahadurshah Zafar Marg, New Delhi, Delhi 110002 India; 11 Division of Rheumatology, Faculdade de Medicina FMUSP, Universidade de Sao Paulo, Sao Paulo, SP Brazil; 12 Southport and Ormskirk Hospital NHS Trust, Southport, PR8 6PN UK; 13 Department of Medicine, Queen Savang Vadhana Memorial Hospital, Chonburi, Thailand; 14 Rheumatology Department, Saint-Joseph University, Beirut, Lebanon; 15 Rheumatology Department, Hotel-Dieu de France Hospital, Beirut, Lebanon; 16 Division of Allergy, Immunology and Rheumatology, Department of Internal Medicine, Taichung Veterans General Hospital, Taichung City, Taiwan; 17 Department of Medical Research, Taichung Veterans General Hospital, Taichung, Taiwan; 18 Department of Medicine, Section of Rheumatology, St. Luke’s Medical Center-Global City, Taguig, Philippines; 19 Reference Center for Osteoporosis, Rheumatology and Dermatology, Pontifica Universidad Javeriana Cali, Cali, Colombia; 20 Department of Rheumatology, National Centre for Rheumatic Diseases, Ratopul, Kathmandu, Nepal; 21 Department of Clinical Immunology and Rheumatology, Sanjay Gandhi Postgraduate Institute of Medical Sciences, Lucknow, India; 22 Department of Rheumatology, Royal Wolverhampton Hospitals NHS Trust, Wolverhampton, UK; 23 Division of Musculoskeletal and Dermatological Sciences, School of Biological Sciences, Faculty of Biology, Medicine and Health, Centre for Musculoskeletal ResearchManchester Academic Health Science CentreThe University of Manchester, Manchester, UK

**Keywords:** Vaccination, Rheumatoid arthritis, COVID-19, Coronavirus, Survey

## Abstract

This study aimed to assess COVID-19 vaccination-related AEs in patients with rheumatoid arthritis (RA), in the COVID-19 Vaccination in Autoimmune Diseases (COVAD)-2 study. An online international cross-sectional survey captured self-reported data on COVID-19 vaccination-related adverse events (AEs) in people with RA, autoimmune diseases (AIDs; rheumatic [r] and non-rheumatic [nr]) and healthy controls (HCs). The survey was circulated by the COVAD study group, comprising 157 collaborators across 106 countries, from February to June 2022. Delayed AEs among RA were compared with other rAIDs, nrAIDs and HCs using multivariable binary regression. A total of 7203 participants were included (1423 [19.7%] RA, 2620 [36.4%] rAIDs, 426 [5.9%] nrAIDs, 2734 [38%] HCs), with 75% female. Compared to HCs, individuals with RA reported higher overall major AEs [OR 1.3 (1.0–1.7)], and an increased number of several minor AEs. Compared to nrAIDs, people with RA had several increased reported minor AEs including myalgia and joint pain. People with active RA had increased major AEs [OR 1.8 (1.1–3.0)] and hospitalisation [OR 4.1 (1.3 – 13.3)] compared to inactive RA. RA patients without autoimmune comorbidities had significantly fewer major and minor AEs than those with other rAIDs. A decreased incidence of hospitalisation was seen in patients taking methotrexate or TNF inhibitors compared to patients not taking these medications. COVID-19 vaccination is associated with minimal to no risks of delayed AEs in patients with RA compared to HCs, and fewer compared to other rAIDs. Active RA and presence of co-existing rAIDs were associated with an increased risk of delayed AEs.

**Supplementary Information:**

The online version contains supplementary material available at 10.1007/s00296-024-05742-x.

## Introduction

Vaccination against coronavirus disease 2019 (COVID-19) has been proven safe in the healthy population [1]. This has led to a significant decrease in the morbidity and mortality associated with COVID-19. However, certain at-risk populations, such as those with immune-mediated inflammatory diseases (IMIDs) and/or immunocompromised individuals, have been deliberately excluded from vaccine trials. In addition, vaccine hesitancy remains among people with IMIDs, due to reasons including fear of flares and side-effects on a background of chronic disease [2]. The same group also has the highest risk of complications arising from COVID-19 [3]. Short-term safety data for COVID-19 vaccination are reassuring in both healthy and high-risk groups, including those with IMIDs [4–8].

Data on delayed adverse events (AEs) occurring greater than seven days post-vaccination, are lacking, especially in people with autoimmune diseases and immunocompromised patients [9]. Our recent results from the COVID-19 Vaccination in Autoimmune Diseases (COVAD) study, within the idiopathic inflammatory myositis (IIM) cohort, demonstrated reassuring data in this regard, with a low incidence of delayed AEs [10].

People with rheumatoid arthritis (RA) are at the highest risk of adverse outcomes, including hospitalisation, intensive care unit admission and mortality, following infection with COVID-19 [11, 12]. Previous results from the COVAD study have demonstrated reassuring data for AEs occurring within seven days of vaccination in individuals with RA, with AE profiles comparable to healthy controls (HC). However, the lack of information on delayed AEs following COVID-19 vaccination could perpetuate vaccine hesitancy in this high-risk group. RA remains the most commonly diagnosed autoimmune rheumatic disease, with many patients on high levels of immunosuppression. On average, people with RA have two or more comorbidities, putting them at increased risk of infections and emphasising the need for effective vaccines in this population. These aspects increase the urgency to understand the response to vaccination, including delayed AEs, in this high-risk group.

In this study, we aimed to assess COVID-19 vaccination-related AEs in people with RA, rheumatic autoimmune diseases (rAIDs), and non-rheumatic AIDs (nrAIDs) and HC, occurring at greater than seven days post-vaccination in the second COVAD (COVAD-2) study.

## Methods

### Study design

This study was undertaken as part of the COVAD-2 study, an ongoing international cross-sectional, multi-centre patient self-reported online survey [9]. This survey follows from the first COVAD survey, of similar format, circulated in 2021, which looked at short-term vaccination-related adverse events. This survey collected meaningful data on the safety and tolerability of the COVID-19 vaccines in patients with IMIDs as well as healthy individuals, through a series of 36 questions. Together with patient partners and experts in multiple fields including rheumatology, neurology and immunology, COVAD-2 was built and widely disseminated. Informed consent from the participants was obtained electronically via an initial question (requesting consent) in the online survey following the opportunity to review an information cover letter. Incentives were not offered to complete the survey. Ethics approval was obtained from the Sanjay Gandhi Postgraduate Institute of Medical Sciences (SGPGIMS) ethics committee (IEC Code: 2021-143-IP-EXP-39) and the Checklist for Reporting results of the Internet E-Surveys was adhered to in the reporting of results [13].

This survey adhered to the latest recommendations for survey drafting and reporting.

### Patient involvement

Patients were involved in the study design of COVAD, including the development of the online questionnaire.

### Data collection

A validated questionnaire was hosted on an online platform (www.surveymonkey.com), following pilot testing, vetting and revision by an international team of experts, and was translated into 18 languages. The survey was circulated extensively by the COVAD study group comprising 157 collaborators across 106 countries in their clinics, patient support groups, and social media platforms from February to June 2022. The full survey has previously been published [9].

Data collected included patient demographics, comorbidities, IMID diagnosis (both rAIDs and nrAIDs), treatment details, current symptom status, COVID-19 infection history, course, and outcomes (including hospitalization and need for oxygen therapy), COVID-19 vaccination details, short-term (< 7 days) and delayed-onset (> 7 days) post-vaccination AEs (based on the Centers for Disease Control and Prevention [CDC] criteria), and patient-reported outcomes according to the Patient Reported Outcomes Measurement Information System (PROMIS) [14]. rAIDs included connective tissue diseases, inflammatory myopathies and inflammatory arthritis. nrAIDs included inflammatory bowel disease, multiple sclerosis etc. Individuals over the age of 18 years, were included in this study. The methods have been previously described in detail [9].

### Data extraction

Data extraction was completed on 10th July 2022, with responses included only from participants who had completed the survey in full and had received at least one dose of any COVID-19 vaccine at the time of completing the survey. Relevant variables were extracted, including self-reported post-vaccination AEs for > 7 days, demographics, clinical characteristics and COVID-19 infection history.

### Active and inactive disease

Participants were able to self-report their disease activity as “inactive/ remission,” “active and improving,” “active but stable,” “active and worsening,” “I am not sure,” or “other”. Self-reported disease status was additionally verified using information collected on symptomology and treatment regimens prior to vaccination.

### Adverse events post-vaccination

Delayed onset AEs were defined as those occurring > 7 days post-vaccination. They were categorised into minor AEs, major AEs requiring medical attention (without hospitalisation) and major AEs requiring hospitalisation [15]. An open-ended question was asked to enable participants to report additional AEs.

### Statistical analysis

Descriptive statistics were presented as median (interquartile ranges; IQR). Individuals with RA were excluded from the rAIDs group, and compared for post-vaccination AEs between active and inactive diseases, as per the definition above. People with RA were compared with other rAIDs, nrAIDs and HCs. Individuals with RA and autoimmune comorbidities were compared with those without comorbidities. Autoimmune comorbidities included other rAIDs and nrAIDs. χ^2^ and Mann–Whitney-*U* tests were used for comparisons between groups for categorical and continuous variables, respectively. Variables that differed across RA, other rAIDs, nrAIDs and HCs in the univariate analysis were included in multivariable binary logistic regression analysis with adjustment for covariates defined a priori- age, sex and ethnicity. The determinants were: the vaccine subtypes, type of immunosuppression, activity of RA, and comorbidity (rAID/nrAID). Stepwise forward regression methodology, guided by both clinical and statistical relevance, was used to identify the most robust regression models. In the multivariable models, statistical significance was set at two-sided P < 0.05. Results were reported as odds ratios (OR) with 95% confidence intervals (CI). Statistical analyses were performed using SPSS version 26 (IBM Corp., Armonk, NY, USA).

## Results

### Baseline characteristics

A total of 17,612 respondents undertook the survey, of whom 7203 with complete responses were included in the analysis. The included participants had a median age of 44 years (34–56), with 75% female and 42.2% Caucasian (Supplementary Table 1). The study cohort comprised 1423 (19.7%) people with RA, 2620 (36.4%) with rAIDs, 426 (5.9%) with nrAIDs and 2734 (38%) HCs. People with RA were older [median age of 51 (40–62)] whereas individuals with rAIDs had 48 (37–59); nrAIDs had 43 (34–53); and HC had 38 (30–49)].

### Post-COVID-19 vaccination-associated AEs in people with RA, compared to other rAIDs, nrAIDs and HCs

Overall, when compared to HCs, people with RA reported major AEs more frequently in the multivariable analyses [OR 1.3 (1.0–1.7)], especially throat closure [OR 2.9 (1.1–7.3)]. Several minor AEs were also reported more frequently in the RA group, including swelling of the extremities [OR 3.7 (1.8–7.7)] and bleeding or bruising [OR 3.2 (1.2–8.5)] (Table 1). People with RA reported episodes of fever less frequently than HCs [OR 0.7 (0.5–1.0)].

**Table 1:** Effects of COVID-19 vaccination in patients with rheumatoid arthritis (RA) vs healthy controls (HCs)

	RA		HCs		Univariable		Multivariable	
	N 1423	% 100	N 2734	% 100	OR (95% CI)	P value	OR (95% CI)	P value
Minor ADEs	211	14.8	456	16.7		0.122		0.499
Injection site (arm) pain and soreness	124	8.7	305	11.2	**0.8 (0.6–0.9)**	**0.014**		0.065
Myalgia	91	6.4	164	6.0		0.615		
Body ache	111	7.8	176	6.4		0.101		0.354
Joint pain	113	7.9	120	4.4	**1.9 (1.4–2.4)**	** < 0.001**	**1.8 (1.3–2.5)**	** < 0.001**
Fever	79	5.6	205	7.5	**0.7 (0.6–0.9)**	**0.018**	**0.7 (0.5–1.0)**	**0.048**
Chills	63	4.4	127	4.6		0.748		
Cough	24	1.7	34	1.2		0.249		
Difficulty in breathing or Shortness of breath	26	1.8	33	1.2		0.109		0.184
Nausea/vomiting	34	2.4	36	1.3	**1.8 (1.1–2.9)**	**0.011**		0.170
Headache	91	6.4	142	5.2		0.111		0.321
Rash	25	1.8	21	0.8	**2.3 (1.3–4.1)**	**0.004**	**2.3 (1.1–4.5)**	**0.022**
Fatigue	111	7.8	136	5.0	**1.6 (1.2–2.1)**	** < 0.001**	**1.5 (1.1–2.0)**	**0.018**
Diarrhoea	25	1.8	33	1.2		0.152		0.574
Abdominal pain	20	1.4	16	0.6	**2.4 (1.3–4.7)**	**0.007**	**2.6 (1.2–5.7)**	**0.018**
High pulse rate or palpitations	26	1.8	45	1.6		0.670		
Rise in blood pressure	16	1.1	19	0.7		0.151		0.370
Fainting	2	0.1	9	0.3		0.261		
Dizziness	45	3.2	51	1.9	**1.7 (1.1–2.6)**	**0.008**	**1.7 (1.0–2.7)**	**0.040**
Chest pain	23	1.6	29	1.1		0.127		0.223
Swelling in the extremities	24	1.7	17	0.6	**2.7 (1.5–5.1)**	**0.001**	**3.7 (1.8–7.7)**	** < 0.001**
Weakness and tingling in the feet and legs	32	2.2	49	1.8		0.313		
Pricking or pins and needles in the hands and feet	25	1.8	28	1.0	**1.7 (1.0–3.0)**	**0.046**		0.232
Visual disturbances (loss of vision, blurring of vision, etc.)	18	1.3	21	0.8		0.115	**2.4 (1.1–5.0)**	**0.022**
Bleeding/bruising on the body	13	0.9	9	0.3	**2.8 (1.2–6.5)**	**0.014**	**3.2 (1.2–8.5)**	**0.024**
Petechial rash	6	0.4	5	0.2		0.155		0.254
Major ADEs	159	11.2	224	8.2	**1.4 (1.1–1.7)**	**0.002**	**1.3 (1.0–1.7)**	**0.046**
Anaphylaxis	9	0.6	19	0.7		0.815		
Marked difficulty in breathing	22	1.5	42	1.5		0.982		
Throat closure	12	0.8	13	0.5		0.146	**2.9 (1.1–7.3)**	**0.029**
Severe rashes	17	1.2	29	1.1		0.696		
Hospitalisation	52	3.7	57	2.1	**1.8 (1.2–2.6)**	**0.003**		0.592

Outcomes which are significant are highlighted in bold

Factors included as covariates in multivariable binary logistic regression analysis included age, sex, ethnicity

Compared to rAIDs, people with RA reported rash [OR 0.6 (0.4–1.0)] less frequently; however, all other major and minor AEs were comparable in the multivariable binary logistic regression model (Table 2).

**Table 2:** Effects of COVID-19 vaccination in patients with rheumatoid arthritis (RA) and no autoimmune comorbidities vs RA with rheumatic autoimmune disease (rAIDs)

	RA without AIDs		RA + rAID		Univariable		Multivariable	
	N (965)	100%	N (334)	100%	OR (95% CI)	P value	OR (95% CI)	P value
Minor ADEs	126	13.1	69	20.7	**0.6 (0.4–0.8)**	**0.001**	**0.6 (0.4–0.8)**	**0.001**
Injection site (arm) pain and soreness	71	7.4	41	12.3	**0.6 (0.4–0.9)**	**0.006**	**0.6 (0.4–0.8)**	**0.005**
Myalgia	53	5.5	28	8.4	**-**	0.060		0.079
Body ache	65	6.7	39	11.7	**0.5 (0.4–0.8)**	**0.004**	**0.5 (0.4–0.8)**	**0.006**
J oint pain	66	6.8	37	11.1	**0.6 (0.4–0.9)**	**0.013**	**0.6 (0.4–0.9)**	**0.017**
Fever	46	4.8	27	8.1	**0.6 (0.4–0.9)**	**0.023**	**0.6 (0.4–1.0)**	**0.043**
Chills	34	3.5	23	6.9	**0.5 (0.3–0.9)**	**0.010**	**0.5 (0.3–0.8)**	**0.010**
Cough	10	1.0	12	3.6	**0.3 (0.1–0.7)**	**0.002**	**0.3 (0.1–0.7)**	**0.004**
Difficulty in breathing or Shortness of breath	12	1.2	10	3.0	**0.4 (0.2–1.0)**	**0.033**		0.089
Nausea/vomiting	18	1.9	13	3.9	**0.5 90.2–1.0)**	**0.036**	**0.5 (0.2–1.0)**	**0.039**
Headache	47	4.9	37	11.1	**0.4 (0.3–0.6)**	** < 0.001**	**0.4 (0.3–0.7)**	** < 0.001**
Rash	13	1.3	11	3.3	**0.4 (0.2–0.9)**	**0.023**	**0.4 (0.2–0.9)**	**0.021**
Fatigue	54	5.6	47	14.1	**0.4 (0.2–0.5)**	** < 0.001**	**0.4 (0.2–0.6)**	** < 0.001**
Diarrhoea	13	1.3	11	3.3	**0.4 (0.2–0.9)**	**0.023**	**0.4 (0.2–0.9)**	**0.029**
Abdominal pain	8	0.8	9	2.7	**0.3 (0.1–0.8)**	**0.010**	**0.3 (0.1–0.8)**	**0.017**
High pulse rate or palpitations	12	1.2	11	3.3	**0.4 (0.2–0.8)**	**0.014**	**0.4 (0.2–1.0)**	**0.041**
Rise in blood pressure	9	0.9	6	1.8	–	0.203		
Fainting	1	0.1	1	0.3	–	0.432		
Dizziness	25	2.6	17	5.1	**0.5 (0.3–0.9)**	**0.026**		0.053
Chest pain	8	0.8	11	3.3	**0.2 (0.1–0.6)**	**0.001**	**0.3 (0.1–0.7)**	**0.005**
Swelling in the extremities	13	1.3	9	2.7	-	0.100		0.133
Weakness and tingling in the feet and legs	18	1.9	13	3.9	**0.5 (0.2–1.0)**	**0.036**		0.099
Pricking or pins and needles in the hands and feet	16	1.7	8	2.4	–	0.389		
Visual disturbances (loss of vision, blurring of vision, etc.)	7	0.7	10	3.0	**0.2 (0.1–0.6)**	**0.002**	**0.3 (0.1–0.7)**	**0.008**
Bleeding/bruising on the body	7	0.7	5	1.5	–	0.204		
Petechial rash	2	0.2	4	1.2	**0.2 (0.03–0.9)**	**0.021**	**0.2 (0.03–0.9)**	**0.034**
Major ADEs	96	9.9	48	14.4	**0,7 (0.5–1.0)**	**0.026**	**0.7 (0.5–1.0)**	**0.032**
Anaphylaxis	2	0.2	6	1.8	**0.1 (0.02–0.6)**	**0.001**	**0.1 (0.02–0.6)**	**0.009**
Marked difficulty in breathing	8	0.8	9	2.7	**0.3 (0.1–0.8)**	**0.010**	**0.3 (0.1–0.8)**	**0.015**
Throat closure	3	0.3	7	2.1	**0.1 (0.03–0.6)**	**0.001**	**0.1 (0.03–0.6)**	**0.005**
Severe rashes	8	0.8	9	2.7	**0.3 (0.1–0.8)**	**0.010**	**0.3 (0.1–0.8)**	**0.012**
Hospitalisation	32	3.3	16	4.8	**–**	0.219		

Outcomes which are significant are highlighted in bold

Factors included as covariates in multivariable binary logistic regression analysis included age, sex, ethnicity

Compared to nrAIDs, people with RA more frequently reported injection site pain [OR 1.7 (1.0–2.6)], myalgia [OR 1.9 (1.1–3.4)], body ache [OR 2.2 (1.3–3.7)], joint pain [OR 2.7 (1.5–4.8)], fever [OR 1.8 (1.0–3.3)] and swelling of the extremities [OR 4.9 (1.1–21.4)]. No significant difference was found in the frequency of reporting major AEs between people with RA and nrAIDs.

### Post-COVID-19 vaccination-associated AEs across vaccine subtypes and by type of immunosuppression

Among people with RA, 69.2% (n = 986) received the BNT162b2 (Pfizer) vaccine, 36.3% (n = 517) received ChadOx1 nCOV-19 (Oxford/ AstraZeneca), 17.8% (n = 254) received mRNA-1273 (Moderna) and 7.9% (n = 112) received Sinovac-CoronaVac. Of note, some patients had received more than one dose of more than one type at the time of completing the survey, accounting for these data. ChadOx1 nCOV-19 (Oxford/ AstraZeneca) led to a significantly increased frequency of reported minor AEs in the RA group [OR 1.9 (1.4–2.6)], compared to other vaccines (Supplementary Table 2). Moderna vaccination was associated with an increased frequency of reported hospitalisation in people with RA [β 2.4 (1.3–4.3)].

In the multivariable binary logistic regression, people with RA who received ChadOx1 nCOV-19 (Oxford/ AstraZeneca) had a significantly higher frequency of reporting several minor AEs compared to all other vaccines, including pins and needles in hands and feet [OR 3.0 (1.3–6.9)], bleeding or bruising [OR 5.2 (1.4–19.5)] (Supplementary Table 2).

A decreased frequency of reporting of hospitalisation was seen in patients taking methotrexate [OR 0.5 (0.3–0.9)] or TNF inhibitors [OR 0.1 (0.02–0.9)] compared to patients not taking these medications. Major AEs were also less frequently reported in patients taking methotrexate, compared to those not taking methotrexate [OR 0.7 (0.5–1.0)]. People with RA taking leflunomide had an increased frequency of reporting swelling in the extremities compared to patients not taking leflunomide [OR 3.0 (1.1–7.7)].

### Post-COVID-19 vaccination-associated AEs in people with active and inactive RA

People with active RA had an increased frequency of reporting major AEs [OR 1.8 (1.1–3.0)] and hospitalisation [OR 4.1 (1.3–13.3)], compared to inactive RA. Of note, people with active RA less frequently reported palpitations [OR 0.4 (0.2–1.0)] and [OR 0.3 (0.1–0.9)] hypertension > 7 days post-vaccination, compared to those with inactive disease.

### Post-COVID-19 vaccination-associated AEs in people with only RA, RA and rAID comorbidity and RA with nrAID comorbidity

Individuals with RA alone reported major and minor AEs less frequently compared to those with other rAIDs, across almost all domains (Table 2). Compared to people with RA and nrAID comorbidities, people with RA alone reported throat closure [OR 0.1 (0.02–0.9)] less frequently, whereas no significant difference was found between the two groups for all minor AEs and all other major AEs.

People with RA and mental health diagnoses had increased reported chills [OR 1.8 (1.1–3.0)] and chest pain [OR 2.5 (1.1–6.0)] than those with no reported mental health disorders (Supplementary Table 3). No significant difference was found between the two groups regarding major AEs. Mental health disorders included anxiety, bipolar disorder, depression, eating disorders, insomnia, schizophrenia and substance use disorders.

## Discussion

This study provides insights into delayed AEs at greater than seven days after the administration of the COVID-19 vaccination in people with RA. This is the first study to assess delayed AEs associated with COVID-19 vaccination in this patient cohort. This is also the first study to compare people with RA with those with other autoimmune diseases and HCs. While our study demonstrated that active RA and comorbid rAIDs increased the likelihood of AEs, and an overall greater incidence of minor AEs compared to HCs, the data is reassuring, with minimal to no risks of delayed AEs in this cohort, comparable to those with other rAIDs.

These findings are consistent with those of other studies on AEs following COVID-19 vaccination in people with rAIDs, although few have examined delayed AEs. In our study, 22.6% of people with RA reported AEs at > 7 days post-vaccination. Results from the EULAR Coronavirus Vaccine (COVAX) study, where AEs were reported by clinicians, found one third of patients experienced AEs, although these were at < 7 days post-vaccination [16]. However, delayed AEs have been studied in people with IIM within the COVAD-2 cohort, demonstrating a similar safety profile with low overall risk of both minor and major AEs at > 7 days and rare episodes of hospitalisations [10].

An important point to consider is the similarity between minor AEs and symptoms associated with active RA, particularly given the self-reported nature of the data. A pertinent example is the higher incidence of joint pain, fatigue and swelling of the extremities in people with RA compared with HCs. Swelling of extremities was significantly higher in the RA population than HCs and nrAIDs—this may have been interpreted by certain patients as joint swelling, especially when translated across languages. However, it is important to note that studies to date have found no association between COVID-19 vaccination and RA flares, similar to work on previous vaccinations [17, 18]. It is reassuring that people with RA had similar reported incidence of major AEs compared to rAIDs and nrAIDs, with only a slightly increased number reported compared to HCs. However, major AEs were more likely to be reported in people with active RA than in those with inactive RA, which may be associated with a greater degree of inflammation and a clinically vulnerable state.

A significantly greater number of minor AEs were reported in people with RA receiving the ChadOx1 nCOV-19 (Oxford/ AstraZeneca) vaccination, similar to our findings in the IIM cohort, when studying delayed AEs [10]. In contrast, in our previous study investigating short-term AEs in people with RA, recipients of the mRNA-1273 (Moderna) vaccine had higher frequencies of vaccine-related AEs [6]. However, in the present study, recipients of the Moderna vaccine had significantly higher rates of hospitalisation than recipients of all other vaccinations.

There was a decreased frequency of hospitalisation in patients taking methotrexate and/or TNF inhibitors compared to those patients not taking these medications. Major AEs were also less frequent in patients taking methotrexate than those taking all other included DMARDs. It should be noted that, depending on local and international guidance at the time of vaccine administration, patients with IMIDs, including RA, were advised to withhold their drug up to 2–4 weeks before and/or after vaccination, particularly true of methotrexate and anti-TNF medications [19–21]. However, it is not possible to know, from these self-reported data, whether or not patients had held their DMARD at the time of vaccination. Vaccination was advised, where possible, prior to starting a new immunomodulatory therapy. These guidelines aimed primarily to boost the immune response resulting from vaccination, as demonstrated for methotrexate in a randomised, open-label superiority trial [22]. However, there is a paucity of data regarding stopping these drugs peri-vaccination, and if this may influence subsequent early and delayed AEs, and is certainly something that needs to be taken into consideration, especially given the self-reported nature of these data.

Our study had some limitations. Data were entirely self-reported by patients, without verification of medical records or by healthcare practitioners. The fact that the survey was internet-based may have led to selection bias, potentially excluding people in regions with poor internet connectivity, lower socioeconomic status, and those with impaired use or access to technologies. Patients were not explicitly questioned about flares of RA following vaccination at > 7 days, but on symptoms that may have overlapped with this, making it difficult to deduce if reported symptoms were secondary to vaccination, flare, or both. However, it should be noted that flares of RA following vaccination and humoral responses have been documented elsewhere, and were not within the scope of this study. Furthermore, the large number of patients taken from a global sample, comprising patients with early and established RA and active and inactive disease, represents an important strength. Self-reported data also champions the patients’ voice and removes potential biases introduced through data collection by a health professional, especially regarding subjective symptoms such as pain, and when discussing potentially sensitive topics such as mental health disorders.

Future directions from this study include further exploration on the intersection of autoimmunity potentially associated with vaccination and self-reported symptoms which were already present due to impending flare. Indeed, one piece of work arising from the COVAD-2 study suggested that the group most likely to flare after vaccination was those who were already experiencing mild rash and arthritis or muscle weakness [23]. Another potential future study would be identification of the group reporting symptoms in the presence of autoimmune multimorbidity, arising out of heightened perception to pain and fatigue, and may not necessarily be non-inflammatory.

In conclusion, our findings in this international cohort of people with RA, compared with HCs and individuals with other rAIDs and nrAIDs, are reassuring in terms of COVID-19 vaccination in this patient population, with regard to the onset of AEs > 7 days post-vaccination. While certain AEs were more frequently reported in individuals with RA compared to HCs, these were minor in most cases. Our findings are expected to encourage vaccination in this high-risk group of patients, especially given the overall reassuring data in people taking various forms of immunosuppression and those with both active and inactive diseases. The detailed observations in these patient-self-reported data have the potential to contribute to future vaccination guidance as the pandemic enters a new phase, identifying subgroups of individuals who may require special considerations or monitoring. Above all, we hope that the findings of this study will help to overcome vaccine hesitancy and, ultimately, adverse outcomes of COVID-19.

## Supplementary Information

Below is the link to the electronic supplementary material.

Supplementary file1 (DOCX 57 KB)

Supplementary file2 (DOCX 17 KB)

## Data Availability

The datasets generated and/or analyzed during the current study are not publicly available but are available from the corresponding author upon reasonable request.
