# Supplementary material for: COVID-19 vaccination-related delayed adverse events among people with rheumatoid arthritis: results from the international COVAD survey
Source: Rheumatol Int. 2024 Nov 6;44(12):2853–61. doi: 10.1007/s00296-024-05742-x (PMC11618185; doi:10.1007/s00296-024-05742-x)
Supplement: Supplementary file 2 — Supplementary file2 (DOCX 17 KB) [file 296_2024_5742_MOESM2_ESM.docx]

| **Variable** | **Total, n (7203)** | **(%) (100)** | **RA,**  **n (1423)** | **%**  **(19.7)** | **rAIDs, n (2620)** | **% (36.4)** | **nrAIDs,**  **n (426)** | **% (5.9)** | **HC,**  **n (2734)** | **% (38)** |  |
| --- | --- | --- | --- | --- | --- | --- | --- | --- | --- | --- | --- |
| **Age (Me, IQR), years** | 44 (34-56) | - | 51 (40-62) | - | 48 (37-59) | - | 43 (34-53) | - | 38 (30-49) | - |  |
| **Sex (F:M)** | 5310:1799  3:1 | - | 1192:210  5.7:1 | - | 2155:438  5:1 | - | 351:70  5:1 | - | 1611:1081  1.5:1 | - |  |
| **Ethnicity n (%)** | | | | | | | | | | | |
| African American or of African origin (Black) | 336 | 4.7 | 49 | 3.4 | 186 | 7.1 | 4 | 0.9 | 97 | 3.5 |  |
| Asian | 1632 | 22.7 | 322 | 22.6 | 542 | 20.7 | 57 | 13.4 | 710 | 26.0 |  |
| Caucasian (White) | 3039 | 42.2 | 784 | 55.1 | 1274 | 48.6 | 227 | 53.3 | 754 | 27.6 |  |
| Do not wish to disclose | 262 | 3.6 | 49 | 3.4 | 84 | 3.2 | 14 | 3.3 | 115 | 4.2 |  |
| Hispanic | 1200 | 16.7 | 104 | 7.3 | 293 | 11.2 | 83 | 19.5 | 720 | 26.3 |  |
| Native American/Indigenous/Pacific Islander | 53 | 0.7 | 8 | 0.6 | 21 | 0.8 | 4 | 0.9 | 20 | 0.7 |  |
| Other | 628 | 8.7 | 98 | 6.9 | 206 | 7.9 | 34 | 8.0 | 290 | 10.6 |  |

Supplementary Table 1: Socio-demographic and vaccination data of survey respondents. RA: rheumatoid arthritis; rAID: rheumatic autoimmune disease; nrAID: non-rheumatic autoimmune disease; HC: health controls.
